# Insulin Receptor Signaling in Health and Disease

**DOI:** 10.3390/biom13050807

**Published:** 2023-05-09

**Authors:** Leili Baghaie, David A. Bunsick, Myron R. Szewczuk

**Affiliations:** Department of Biomedical and Molecular Sciences, Queen’s University, Kingston, ON K7L 3N6, Canada; 16lbn1@queensu.ca (L.B.); 17dab5@queensu.ca (D.A.B.)

Since the discovery of insulin over 100 years ago, our understanding of the insulin signaling pathway has greatly expanded. The insulin receptor (IR) is now better recognized as the signaling protein that regulates metabolism through cell differentiation and growth. Following IR-β kinase activity, the downstream effects of this signaling paradigm are vital for the glucose homeostasis of many cell types. However, this process requires balance, as increased insulin levels lead to decreased insulin sensitivity and, eventually, resistance. The clinical implications of insulin resistance include cancer, type 2 diabetes (metabolic syndrome), hypertension, and cardiovascular disease. Although it is suggested that insulin resistance occurs due to the downregulation of the IR, or insulin receptor substrate (IRS) proteins, the precise mechanism of insulin resistance is unknown. In this Special Issue of *Biomolecules*, the critical players in regulating the insulin pathway are discussed, considering the external factors involved in the onset of disease.

It is well-accepted that diet plays a vital role in insulin and fat metabolism pathways. Most literature focuses on the nutrients that may make an individual more susceptible to insulin resistance; however, more recently, certain studies are interested in the protective effects of some diets. For example, in a study by Tong et al. [1], dietary soy reduced alcohol-mediated neurocognitive dysfunction and improved brain insulin pathway signaling in adolescent rats. Adolescents are highly susceptible to ethanol-mediated sustained neurocognitive dysfunction for diseases such as alcohol-related brain disease (ARBD), which results in cognitive–motor deficits and a potential progression to dementia and disability. The substitution of soy isolate resulted in less insulin signaling impairment and better cognitive function when exposed to alcohol. In contrast, it is suggested that vitamin A (VA) plays an essential role in the development of type 2 diabetes (T2D) and obesity in Zucker diabetic fatty (ZDF) rats. In a study by Wang et al. [2], rats fed a diet low in vitamin A displayed decreased body weight gain and an improved sensitivity to insulin and glucose tolerance. Furthermore, a low VA diet also reduced the expression of genes linked to lipogenesis and adipogenesis. Together, these studies demonstrate that dietary interventions, such as the restriction of vitamin A and the implantation of a soy-rich diet, may prevent the onset of insulin resistance; however, these results must first be replicated in humans.

It is of interest to further investigate how diet can alter insulin metabolism at a molecular level. Advanced glycated end-products (AGEs), which Khalid et al. [3] discuss in their review, arise through exogenous and endogenous sources, such as food and the glycan process, through a non-enzymatic reaction between sugars and proteins, lipids, or nucleic acids. Their accumulation is associated with insulin resistance, oxidative stress, and inflammation. They function by trapping and cross-linking proteins or indirectly binding to the cell surface receptor, allowing them to signal through several receptor pathways. These pathways include the mitogen-activated protein kinase (MAPK) and Janus kinase signal transducer and activator of transcription (JAK/STAT) pathway, which have previously been linked to insulin resistance and diabetes. AGEs can also accumulate in the pancreas contributing to beta-cell toxicity via an inflammatory cascade and oxidative stress activation. Additionally, several studies found that using the soluble receptor for advanced glycation end-products (sRAGEs) preserves beta-cell morphology and blocks inflammatory mediators, indicating a potential therapeutic intervention in chronic diabetes.

Aside from diet as a potential therapeutic, a critical biomarker that may aid in diagnoses is discussed in this Special Issue. In a patient study, Simonienė et al. [4] noted that the serum expression of miR-107, a critical mRNA in glucose metabolism, can be used as a marker of the severity of type 2 diabetes (T2D) and its associated risk factors. In this study, the expression of miR-107 positively correlated with BMI, age, and the insulin resistance index (HOMA-IR). These findings extended to individuals with advanced diabetes who also had higher levels of miR-107 than non-diabetic overweight individuals, with median serum miR-107 levels of 1.33 and 0.63, respectively. Moreover, a higher expression was found in insulin and metformin users. Contrastingly, miR-107 levels negatively correlated with HDL, further demonstrating that it can potentially be a biomarker for type 2 diabetes. A better understanding of insulin’s role in the body will significantly impact on the development of insights for this disease.

The impact of insulin resistance on the body’s organs is vast and has been implicated in many pathological conditions. The heart, for example, expresses abundant insulin receptors and is considered an insulin-dependent organ that regulates cardiac metabolism and contractility in cardiomyocytes, fibroblasts, and endothelial cells. Pantazi et al. [5] reviewed how the loss of the vital insulin signaling receptor substrates 1 and 2 (IRS1 and IRS2) is linked to insulin resistance and heart failure (HF). In both type 1 and type 2 diabetes mellitus (DM) patients, the workload of the left ventricle often increases, making heart failure an early sign of DM. Moreover, in both HF and diabetes, an increase in insulin levels has been noted, leading to constantly stimulating insulin receptors. Many patients suffering from HF acquire left ventricular assist devices (LVADs), which bypass the failing left ventricle, allowing for the heart to pump blood to the rest of the body. One of the associated outcomes of the implantation of LVADs is the increased blood flow to the pancreas, reducing insulin resistance and decreasing fasting blood glucose levels in diabetic and non-diabetic patients. It is believed that the latter results from the increased expression of the insulin-independent glucose transporter type 4 (GLUT4), seen in most patients with LVADs.

The disruption of proper signaling significantly impacts the heart, and more recent findings demonstrate that it may also play a more prominent role in conditions not previously associated with insulin signaling. As such, the review by Patel et al. [6] discusses the insulin effects on the striatum region of the brain. The striatum region has a significant role in motor control, reward, and addiction, and has a high expression of insulin receptors. The review discusses insulin’s potential to regulate dopamine release and reward-related behaviors in the brain. It postulates that insulin receptors (IRs) activating cholinergic interneurons (Chls) enhance Chls to boost ACh-mediated dopamine release. Moreover, uncovering insulin’s role in the striatum suggests that it may play a role in modulating reward-related behaviors such as addiction and feeding. There is some evidence that insulin resistance may have a role in age-related neurodegenerative disorders, including Parkinson’s (PD) and Alzheimer’s (AD). Previous literature has indicated associations between type 2 diabetes and PD, as studies have shown PD pathology and symptoms appearing faster and more severely in the type 2 diabetes group. Several studies have found that insulin-resistant mice overexpressing the PED/PEA-15 protein exhibit metabolic changes in the striatum and decreased striatal TH expression and dopamine content. The evidence suggests that insulin may significantly affect brain function beyond its well-known role in glucose metabolism and have more significant implications in other diseases, including AD and PD.

Together, the findings from the papers listed in this Special Issue of *Biomolecules* demonstrate that we have come a long way from the discovery of insulin over 100 years ago. However, there is still information left to be uncovered. Aside from its well-known impact on diabetes, other pathological conditions, including cardiovascular disease and neurodegenerative disorders, are shown to be implicated in insulin signaling. Moreover, new methods to prevent and diagnose the associated risk factors of insulin resistance were discussed. The goal was to provide an overview of our current understanding of the field of insulin that can be expanded on for future research involving potential therapeutics.
